# Myocardial perfusion imaging in coronary artery disease: SPECT, PET or CMR?

**DOI:** 10.1007/s12471-012-0300-z

**Published:** 2012-07-03

**Authors:** E. E. van der Wall

**Affiliations:** 1 Interuniversity Cardiology Institute of the Netherlands (ICIN) - Netherlands Heart Institute (NHI), Utrecht, the Netherlands; 2 Department of Cardiology, Leiden University Medical Center, Albinusdreef 2 Postal zone: K5-35, P.O. Box 9600, 2300 RC Leiden, the Netherlands

Noninvasive imaging in the evaluation of a wide variety of cardiovascular diseases has gained an increasing role in the diagnostic strategy in current cardiology practice [1–4]. This holds in particular for patients with myocardial ischaemia due to coronary artery disease (CAD) [5–7]. Of the present imaging modalities, single-photon emission computed tomography (SPECT), positron emission tomography (PET) and cardiac magnetic resonance (CMR) have attained a major position when it comes to myocardial perfusion imaging [8–12].

In the May 2012 issue of the Journal of the American College of Cardiology (JACC), Jaarsma et al. [13] from Maastricht University Medical Center evaluated the diagnostic accuracy of SPECT, PET and CMR for the diagnosis of obstructive CAD. Studies published between 1990 and 2010 identified by PubMed search and citation tracking were examined. A study was included if a perfusion imaging modality was used as a diagnostic test for the detection of obstructive CAD and coronary angiography as the reference standard (50 % diameter luminal stenosis).

Out of a total of 3635 studies, 166 articles (including 17,901 patients) met the inclusion criteria: 114 SPECT, 37 CMR, and 15 PET studies. There were insufficient publications on perfusion echocardiography and computed tomography to include these modalities in the study. Patient-based analysis per imaging modality demonstrated pooled sensitivities of 88 % for SPECT, 84 % for PET , and 89 % for CMR; pooled specificities were 61 %, 81 %, and 76 %, respectively.

The authors concluded that SPECT, PET, and CMR all yielded high sensitivities, whereas a broad range of specificities were observed. CMR and especially PET showed a significantly higher diagnostic accuracy than SPECT. However, SPECT is more widely available, less expensive, and most extensively validated. In addition, the use of attenuation programs improved the specificity of SPECT. CMR may provide a valid alternative without ionising radiation to the nuclear imaging methods. The authors suggested that referring physicians should consider these findings in the context of local expertise and internal logistics.

The authors should be complimented for performing this impressive research. It is the first meta-analysis that has directly compared the three most commonly used techniques for myocardial perfusion imaging, i.e. SPECT, PET, and CMR. The present study emphasises that, from a clinical perspective, each of the studied imaging modalities is in principle suited for detection of abnormalities in myocardial perfusion imaging [14]. As always, selective use is mostly dependent on the institutional availability of the imaging device(s), familiarity with the technique, and the individual expert knowledge of the treating physician [15].
